# Attitude and practice of physical activity and social problem-solving ability among university students

**DOI:** 10.1186/s12199-017-0625-8

**Published:** 2017-04-04

**Authors:** Toshimasa Sone, Yousuke Kawachi, Chihiro Abe, Yuki Otomo, Yul-wan Sung, Seiji Ogawa

**Affiliations:** 1 Department of Rehabilitation, Faculty of Health Science, Tohoku Fukushi University, 1-8-1 Kunimi, Aoba-ku, Sendai, 981-8522 Japan; 2 Kansei Fukushi Research Institute, Tohoku Fukushi University, Sendai, Japan; 3 Department of Welfare Psychology, Graduate School of General Welfare, Tohoku Fukushi University, Sendai, Japan

**Keywords:** Physical activity, Social problem-solving ability, University students, Exercise

## Abstract

**Background:**

Effective social problem-solving abilities can contribute to decreased risk of poor mental health. In addition, physical activity has a favorable effect on mental health. These previous studies suggest that physical activity and social problem-solving ability can interact by helping to sustain mental health. The present study aimed to determine the association between attitude and practice of physical activity and social problem-solving ability among university students.

**Methods:**

Information on physical activity and social problem-solving was collected using a self-administered questionnaire. We analyzed data from 185 students who participated in the questionnaire surveys and psychological tests. Social problem-solving as measured by the Social Problem-Solving Inventory-Revised (SPSI-R) (median score 10.85) was the dependent variable. Multiple logistic regression analysis was employed to calculate the odds ratios (ORs) and 95% confidence intervals (CIs) for higher SPSI-R according to physical activity categories.

**Results:**

The multiple logistic regression analysis indicated that the ORs (95% CI) in reference to participants who said they never considered exercising were 2.08 (0.69–6.93), 1.62 (0.55–5.26), 2.78 (0.86–9.77), and 6.23 (1.81–23.97) for participants who did not exercise but intended to start, tried to exercise but did not, exercised but not regularly, and exercised regularly, respectively. This finding suggested that positive linear association between physical activity and social problem-solving ability (*p* value for linear trend < 0.01).

**Conclusions:**

The present findings suggest that regular physical activity or intention to start physical activity may be an effective strategy to improve social problem-solving ability.

**Electronic supplementary material:**

The online version of this article (doi:10.1186/s12199-017-0625-8) contains supplementary material, which is available to authorized users.

## Background

Social problem-solving ability involves the higher-level cognitive processes needed to cope appropriately with various stressful problems experienced in life [1, 2]. It can be described as encompassing two components: problem orientation and problem-solving skills. Problem orientation refers to an individual’s awareness and perception of problems, and it performs a motivational function in the problem-solving process. Problem-solving skills are effective coping techniques for dealing with a particular problematic situation. They can be divided into four parts: (1) problem definition and formulation, (2) generation of alternative solutions, (3) decision making, and (4) solution implementation and verification [3]. Effective social problem-solving abilities can contribute to decreased risk of poor mental health, including depression [4–6]. Likewise, among university students, it has been reported that higher social problem-solving ability helps to prevent depression and psychological stress [7, 8].

People can also cope with various stresses related to social problems through the use of alternative coping techniques that regulate negative emotions. Physical activity is regarded as one of these emotion-focused coping techniques [9]. In addition, physical activity has a favorable effect on mental health [10, 11]. There are many studies that have reported on mental health issues in students [12]. In addition, previous studies have reported that regular physical activity has been associated with a lower risk of poor mental health in student populations [13–16].

These previous studies suggest that physical activity and social problem-solving ability can interact by helping to sustain mental health. In addition, physical activity is known to be associated with various cognitive functions such as attention or memory [17–20]. Therefore, there could be a direct association between regular physical activity and social problem-solving.

In the present study, we aimed to determine the association between attitude and practice of physical activity and social problem-solving ability among university students. We hypothesized that engaging in regular physical activity enhances social problem-solving ability. We also sought to determine the linear association between these two characteristics.

## Methods

### Study design and participants

Questionnaire surveys and psychological tests were conducted between July and December 2015. The study population comprised students aged 18 years or older enrolled in Tohoku Fukushi University. We recruited 2750 first- and second-year students to participate in the study using email. A total of 186 students (6.8%) participated in the questionnaire surveys and psychological tests. One participant who did not answer questions about physical activity was excluded, leaving 185 participants (6.7%) for analysis. Tohoku Fukushi University has four faculties (including nine departments), in Welfare, Healthcare, Education, and Management. The mission of these faculties is to deeply inquire into the truth, develop widespread knowledge and specialized skills in their fields, and build meaningful relationships, with the ultimate aim of creating paths to the wellbeing for all mankind.

### Measurements

The questionnaire requested the following information from each participant: age, sex, living situation, body weight, height, sleeping condition, dietary habits, physical activity, social activity, psychological distress, and life stressors. The printed questionnaires was administered to participant.

### Attitude and practice of physical activity

Attitude and practice of physical activity was defined as “undertaking moderate physical activity such as walking, riding a bicycle, or doing household tasks while standing or walking for at least 60 min per day in daily life.” This measure has been used in the National Health and Nutrition Survey, conducted by the Ministry of Health, Labour and Welfare of Japan [21]. The possible responses were as follows “I do not practice physical activity, and I never think about doing so,” “I do not practice physical activity, but I intend to start doing so,” “I try to do physical activity, but have not done it much yet,” “Yes, I practice physical activity, but not regularly,” and “Yes, I practice physical activity and regularly.”

### Social problem-solving ability

The Social Problem-Solving Inventory–Revised (SPSI-R) is a 52-item self-report questionnaire that assesses problem-solving skills [22]. The Japanese version of the SPSI-R, which has been validated previously, uses a 50-item self-report questionnaire [23, 24]. The questionnaire contains five subscales: positive problem orientation (PPO), negative problem orientation (NPO), rational problem solving (RPS) (which has four subcomponents: problem definition and formulation (PDF), generation of alternative solutions (GAS), decision making (DM), and solution implementation and verification (SIV)), impulsivity/carelessness style (ICS), and avoidance style (AS). The number of items on each subscale and a sample item are as follows: PPO, four items, e.g., “I believe I can solve a problem if I try hard enough”; NPO, 10 items, e.g., “I feel afraid when I have a problem to solve”; RPS, 20 items, e.g., “I approach problems from many angles”; ICS, 10 items, e.g., “I go with the first good idea that comes to mind”; AS, six items, e.g., “I avoid thinking about problems.” Higher scores on the PPO and RPS (and its subcomponents) indicate more adaptive problem-solving capacity, whereas higher scores on the NPO, ICS, and AS reflect more a more maladaptive approach to problem solving. Each item is answered on a 5-point rating scale with anchors of 0 (“not at all true of me”) and 4 (“extremely true of me”). Sato et al. indicated adequate internal consistency for the Japanese version of the SPSI-R (Cronbach’s α = 0.89) and all subscales (Cronbach’s α = 0.72 to 0.92). In addition, the test–retest reliability over 3 weeks for all subscales as indicated by Pearson’s *r* was indicated from 0.38 to 0.61 [23]. In this study, Cronbach’s α ranged from 0.61 to 0.90 (SPSI-R, 0.90; PPO, 0.61; NPO, 0.88; RPS, 0.86; PDF, 0.64; GAS, 0.61; DM, 0.62; SIV, 0.77; ICS, 0.77; and AS, 0.75).

### Statistical analyses

First, comparisons between physical activity categories were performed via chi-squared analysis to assess differences in characteristics.

Previous studies did not report a well-accepted cut-point for the SPSI-R [22, 23]. Therefore, we conducted analysis of covariance using continuous variables and logistic regression analysis using median scores.

Second, an analysis of covariance was performed to investigate the significance of differences in the SPSI-R among respondents in the various physical activity categories.

Third, we conducted logistic regression analysis to evaluate the association between attitude and practice of physical activity and social problem-solving. Social problem-solving (median score 10.85) was the dependent variable, whereas the independent variable was physical activity, which was divided into five categories: “do not exercise, and never think about doing so (“I do not practice physical activity, and I never think about doing so”),” “do not exercise but intend to start (“I do not practice physical activity, but I intend to start doing so”),” “try to exercise but do not (“I try to do physical activity, but have not done it much yet”),” “exercise but not regularly (“Yes, I practice physical activity, but not regularly,”),” and “exercise regularly (“Yes, I practice physical activity and regularly”)”. Multiple logistic regression analysis was performed to calculate the odds ratios (ORs) and 95% confidence intervals (CIs) for higher social problem-solving according to these five physical activity categories. Furthermore, we repeated the logistic regression analysis after modifying the dependent variable to each subscale, including PPO, NPO, RPS (PDF, GAS, DM, and SIV taken individually), ICS, and AS. The median scores for each of these variables were as follows: PPO, 12; NPO, 23; RPS, 45; PDF, 13; GAS, 12; DM, 11; SIV, 10; ICS, 19; AS, 12.

Fourth, we estimated the *p* value for the linear trend using the five possible responses regarding physical activity level as a continuous variable (“do not exercise, and never think about doing,” “do not exercise but intend to start,” “try to exercise but do not,” “exercise but not regularly,” and “exercise regularly”).

Fifth, we also conducted stratified analyses according to differences in sex, i.e., we queried whether sex significantly interfered with the association between physical activity and social problem-solving ability. Sex, as the interaction between physical activity and social problem-solving ability, was tested through the addition of cross-product terms to the multivariate-adjusted model.

Sixth, in a supplementary analysis, we conducted multiple Poisson regression analysis to evaluate the association between attitude and practice of physical activity and SPSI-R and subscales and to calculate the rate ratios (RRs) and 95% CIs for higher social problem-solving according to these five physical activity categories.

We considered the following variables to be potential confounders: age in years (continuous variable), sex (men or women), and living alone (yes or no). All statistical analyses were performed using SAS version 9.4 (SAS Inc., Cary, NC, USA). We considered differences at *p* < 0.05 to be statistically significant.

## Results

### Characteristics according to physical activity

As shown in Table 1, participants belonging to the categories of “try to exercise but do not,” “exercise but not regularly,” and “exercise regularly” were less likely to be women or living alone than those who said they never considered exercising. In contrast, the proportion of men and those living alone was similar with regards to those who responded that they never considered exercising, and those who did not exercise but intended to begin exercising.

**Table 1:** Characteristics according to physical activity

Characteristic	Total	Physical activity					*P* value
		Do not exercise			Exercise but not regularly	Exercise regularly	
		And never think about doing so	But intend to start	Try to exercise			
No. of subjects	185	20	43	55	35	32	
Age in years (%)							
18	34.6	30.0	23.3	34.5	48.6	37.5	0.44
19	42.2	50.0	53.5	38.2	31.4	40.6	
20	18.9	15.0	16.3	21.8	17.1	21.9	
21	3.2	5.0	2.3	5.5	2.9	0.0	
22	1.1	0.0	4.7	0.0	0.0	0.0	
Sex (%)							
Men	35.7	20.0	20.9	34.5	48.6	53.1	0.01
Women	64.3	80.0	79.1	65.5	51.4	46.9	
Living alone (%)							
Yes	43.2	50.0	48.8	40.0	40.0	40.6	0.85
No	56.8	50.0	51.2	60.0	60.0	59.4	

*P* values were calculated using a chi-square test

### Differences in SPSI-R and subscales among categories of physical activity

The mean score ± standard deviation (SD) on the SPSI-R was 10.4 ± 2.7 for participants who said they never considered exercising, 10.7 ± 2.0 for participants who did not exercise but intended to start, 10.6 ± 2.1 for participants who tried to exercise but did not, 10.8 ± 2.1 for participants who exercised but not regularly, and 12.3 ± 2.8 for participants who exercised regularly. We found significant differences between the five categories of physical activity with respect to the SPSI-R, at the *p* < 0.01 level. Subscale analyses showed the presence of significant differences in NPO means between the five categories of physical activity (NPO; *p* = 0.03) (Table 2).

**Table 2:** Differences in SPSI-R and subscales among categories of physical activity

	Physical activity					*P* value
	Do not exercise			Exercise but not regularly	Exercise regularly	
	And never think about doing so	But intend to start	Try to exercise			
No. of subjects	20	43	55	35	32	
SPSI-R, mean ± SD	10.4 ± 2.7	10.7 ± 2.0	10.6 ± 2.1	10.8 ± 2.1	12.3 ± 2.8	<0.01
PPO, mean ± SD	11.4 ± 4.1	11.6 ± 2.8	11.7 ± 3.4	10.7 ± 3.1	12.9 ± 3.6	0.11
NPO, mean ± SD	23.3 ± 6.7	23.2 ± 6.9	24.0 ± 7.2	21.5 ± 8.2	18.2 ± 10.0	0.03
RPS, mean ± SD	43.9 ± 12.2	44.6 ± 8.6	45.0 ± 10.0	45.2 ± 8.4	49.8 ± 10.5	0.25
PDF, mean ± SD	12.1 ± 3.2	12.9 ± 2.8	12.4 ± 2.7	12.3 ± 2.5	13.4 ± 2.9	0.35
GAS, mean ± SD	11.2 ± 3.8	12.0 ± 2.9	11.5 ± 2.9	12.1 ± 2.8	13.0 ± 3.1	0.29
DM, mean ± SD	9.9 ± 3.0	10.5 ± 2.9	10.5 ± 2.9	11.0 ± 2.7	12.3 ± 3.7	0.08
SIV, mean ± SD	10.7 ± 4.2	9.3 ± 3.5	10.6 ± 3.7	9.8 ± 3.4	11.1 ± 3.6	0.23
ICS, mean ± SD	19.9 ± 6.6	19.3 ± 5.6	20.0 ± 5.6	19.3 ± 4.4	17.7 ± 6.1	0.42
AS, mean ± SD	14.1 ± 5.2	12.7 ± 3.7	13.1 ± 5.0	12.2 ± 4.1	10.8 ± 5.2	0.06

*Abbreviations*: *AS* avoidance style, *DM* decision making, *GAS* generation of alternative solutions, *ICS* impulsivity/carelessness style, *NPO* negative problem orientation, *PDF* problem definition and formulation, *PPO* positive problem orientation, *RPS* rational problem solving, *SD* standard deviation, *SIV* solution implementation and verification, and *SPSI-R* Social Problem-Solving Inventory–Revised

Analysis of covariance was adjusted for age in years (continuous variable), sex (men or women), and living alone (yes or no)

### Multivariate odds ratio and 95% confidence interval of higher SPSI-R and subscales according to physical activity

As shown in Table 3, the unadjusted analysis indicated that the ORs (95% CI) in reference to participants who answered “I do not practice physical activity, and I never think about doing so” were 2.03 (0.68–6.65), 1.68 (0.58–5.34), 3.11 (1.00–10.60), and 7.00 (2.11–26.13) for participants who did not exercise but intended to start, tried to exercise but did not, exercised but not regularly, and exercised regularly, respectively (*p* value for linear trend < 0.01). This finding remained basically unchanged, even after adjusting for age, sex, and living alone (*p* value for linear trend < 0.01). Thus, a greater frequency of higher SPSI-R scores was found among respondents stating that they tried or intended to exercise but did not, as well as those who exercised regularly or irregularly, when compared to those who had not even considered exercising. With regard to subscales and the subcomponents of RPS, linear associations were observed between physical activity and NPO, DM, and ICS. However, there were no significant linear associations between physical activity and PPO, RPS, PDF, GAS, SIV, or AS.

**Table 3:** Multivariate OR and 95% CI of higher SPSI-R and subscales according to physical activity

	Physical activity					*P* value for linear trend
	Do not exercise			Exercise but not regularly	Exercise regularly	
	And never think about doing so	But intend to start	Try to exercise			
No. of subjects	20	43	55	35	32	
SPSI-R						
No. of subjects with higher SPSI-R	6	20	23	20	24	
Crude OR (95% CI)	1.00 (Ref.)	2.03 (0.68–6.65)	1.68 (0.58–5.34)	3.11 (1.00–10.60)	7.00 (2.11–26.13)	< 0.01
Multivariate adjusted OR (95% CI)	1.00 (Ref.)	2.08 (0.69–6.93)	1.62 (0.55–5.26)	2.78 (0.86–9.77)	6.23 (1.81–23.97)	< 0.01
PPO						
Multivariate adjusted OR (95% CI)	1.00 (Ref.)	1.04 (0.35–3.02)	0.90 (0.32–2.53)	0.87 (0.28–2.68)	1.50 (0.47–4.81)	0.58
NPO						
Multivariate adjusted OR (95% CI)	1.00 (Ref.)	0.74 (0.22–2.28)	0.66 (0.20–1.94)	0.42 (0.12–1.34)	0.27 (0.07–0.88)	0.01
RPS						
Multivariate adjusted OR (95% CI)	1.00 (Ref.)	0.71 (0.24–2.08)	0.93 (0.33–2.64)	0.79 (0.25–2.45)	2.37 (0.73–8.00)	0.10
PDF						
Multivariate adjusted OR (95% CI)	1.00 (Ref.)	1.25 (0.43–3.66)	1.18 (0.42–3.33)	0.73 (0.23–2.26)	1.83 (0.57–5.97)	0.64
GAS						
Multivariate adjusted OR (95% CI)	1.00 (Ref.)	1.18 (0.40–3.49)	0.94 (0.33–2.70)	1.37 (0.44–4.35)	2.66 (0.80–9.16)	0.10
DM						
Multivariate adjusted OR (95% CI)	1.00 (Ref.)	1.01 (0.33–3.13)	1.00 (0.34–3.01)	3.34 (1.03–11.47)	3.67 (1.10–13.10)	< 0.01
SIV						
Multivariate adjusted OR (95% CI)	1.00 (Ref.)	0.46 (0.15–1.34)	0.74 (0.24–2.12)	0.82 (0.25–2.59)	1.61 (0.46–5.64)	0.12
ICS						
Multivariate adjusted OR (95% CI)	1.00 (Ref.)	1.38 (0.45–4.16)	0.94 (0.32–2.68)	0.58 (0.18–1.81)	0.48 (0.14–1.51)	0.04
AS						
Multivariate adjusted OR (95% CI)	1.00 (Ref.)	0.70 (0.22–2.08)	0.60 (0.20–1.70)	0.53 (0.16–1.66)	0.35 (0.10–1.11)	0.07

*Abbreviations*: *AS* avoidance style, *CI* confidence interval, *DM* decision making, *GAS* generation of alternative solutions, *ICS* impulsivity/carelessness style, *NPO* negative problem orientation, *OR* odds ratio, *PDF* problem definition and formulation, *PPO* positive problem orientation, *RPS* rational problem solving, *SIV* solution implementation and verification, and *SPSI-R* Social Problem-Solving Inventory–Revised

The outcomes were higher for SPSI-R, PPO, NPO, RPS, PDF, GAS, DM, SIV, ICS, and AS

Multivariate ORs were adjusted for age in years (continuous variable), sex (men or women), and living alone (yes or no)

The *p* value for the linear trend was estimated using the five categories of physical activity as a continuous variable

We further evaluated sex difference in the association between physical activity and social problem-solving ability (Table 4). There was no significant interaction between physical activity and social problem-solving ability (*p* value for interaction = 0.44).

**Table 4:** Multivariate OR and 95% CI of higher SPSI-R according to physical activity stratified by sex

	Physical activity					*P* value for interaction
	Do not exercise			Exercise but not regularly	Exercise regularly	
	And never think about doing so	But intend to start	Try to exercise			
Men						0.44
No. of subjects	4	9	19	17	17	
No. of subjects with higher SPSI-R	1	5	10	10	15	
Crude OR (95% CI)	1.00 (Ref.)	3.75 (0.33–93.82)	3.33 (0.35–74.36)	4.29 (0.44–97.14)	22.50 (1.92–618.34)	
Multivariate adjusted OR (95% CI)	1.00 (Ref.)	3.85 (0.29–107.39)	3.13 (0.32–71.85)	3.58 (0.35–83.98)	20.60 (1.68–583.23)	
Women						
No. of subjects	16	34	36	18	15	
No. of subjects with higher SPSI-R	5	15	13	10	9	
Crude OR (95% CI)	1.00 (Ref.)	1.74 (0.51–6.52)	1.24 (0.36–4.67)	2.75 (0.69–11.99)	3.30 (0.78–15.54)	
Multivariate adjusted OR (95% CI)	1.00 (Ref.)	1.54 (0.44–5.91)	1.36 (0.39–5.23)	3.22 (0.79–14.62)	3.85 (0.88–18.85)	

*Abbreviations*: *CI* confidence interval, *OR* odds ratio, *SPSI-R* Social Problem-Solving Inventory–Revised

The outcomes were higher for SPSI-R

Multivariate ORs were adjusted for age in years (continuous variable), and living alone (yes or no)

### Multivariate rate ratio and 95% confidence interval of higher SPSI-R and subscales according to physical activity

The multiple adjusted analysis indicated that the RRs (95% CI) in reference to participants who answered “I do not practice physical activity, and I never think about doing so” were 1.57 (0.63–3.91), 1.36 (0.55–3.36), 1.78 (0.71–4.49), and 2.31 (0.93–5.74) for participants who did not exercise but intended to start, tried to exercise but did not, exercised but not regularly, and exercised regularly, respectively. Higher SPSI-R was more frequently observed among participants who exercised regularly than those who did not exercise. This linear association was marginally significant (*p* value for linear trend = 0.05). With regard to the subcomponents of RPS, linear associations were observed between physical activity and DM (*p* value for linear trend = 0.04) (Additional file 1: Table S1).

## Discussion

This study, conducted with Japanese university students, indicated that higher SPSI-R was more frequently observed among participants who exercised regularly, compared to those who answered “I do not practice physical activity, and I never think about doing so”. In addition, we found a statistically significant, positive linear association between physical activity and social problem-solving ability.

Our study indicated that higher SPSI-R was more frequently observed among participants who exercised regularly, as compared with those who answered “I do not practice physical activity, and I never think about doing so”. There have been no reports on the reasons for higher SPSI-R scores in participants who exercised regularly. However, previous studies report that exercise may influence cognitive function by increasing brain blood flow [25, 26]. The possibility of a direct or indirect relationship between brain blood flow and higher cognitive function would require additional psychological and neuroscientific research.

Because social problem-solving ability consists of problem orientation and problem-solving skills [1, 2], we attempted to conduct analyses by subscale. We found significant linear associations between physical activity and NPO, DM, and ICS. The overall positive linear association between physical activity and SPSI-R was mainly attributable to the linear associations between physical activity and these three subscale or subcomponent scores. In addition, greater frequency of higher PPO and RPS (including all subcomponents) and lesser frequency of higher NPO, ICS, and AS were observed in participants who exercised regularly than in those who had not even considered exercising. Considering the findings with respect to the definition provided by D’Zurilla et al. [27], for NPO, higher motivation for physical activity is related to a lesser tendency to view a problem as a significant threat to one’s well-being, to doubt one’s own personal ability to solve problems successfully, or to easily become frustrated and upset when confronted with problems. For DM, greater motivation for physical activity is related to a stronger tendency to anticipate the consequences of different solutions, judge and compare them, and then choose the most effective solution. For ICS, greater motivation for physical activity is related to a weaker tendency to act impulsively on the first idea that comes to mind; to scan alternative solutions and consequences quickly, carelessly, and unsystematically; or to monitor solution outcomes carelessly and inadequately. Overall, we found that physical activity was associated with a positive result on the SPSI-R as well as on subscales of the SPSI-R.

Overall, 17.3% of the participants in this study indicated that they exercised regularly, and another 18.9% said that they exercised but not regularly. This means that only 36.2% did at least the relatively minimal amount of exercise defined as physical activity on this questionnaire. According to another study, the proportions of Japanese men and women age 20 to 29 engaging in regular exercise were 16.3% and 16.8%, respectively [28]. Because, in that Ministry of Health study, regular exercise was similarly defined as 30 min of physical activity at least twice a week in accordance with “Health Japan 21” [28, 29], it appears that our study participants were more likely to have regular physical activity than the average among all Japanese age 20 to 29. On the other hand, previous studies in the United States indicated that 39.1% of respondents engaged in at least moderate activity [30] and that 49.9% of students reported moderate to vigorous activity [31]. Therefore, our study participants were less likely to have a high level of physical activity than U.S. students.

The SPSI-R was developed to assess social problem-solving skills [22], and it has been translated in Spanish, Chinese, and Japanese [23, 32, 33]. Among our students, the mean on the SPSI-R was 10.9. Previous studies reported means of 12.0 [23], 11.9 [34], and 12.6 [35]. Thus, our average SPSI-R score was observed to be lower than that of students in previous studies. As our students were recruited from one university without random sampling, our study may demonstrate selection bias. Participants in previous studies were selected from various U.S. universities using random sampling. Therefore, further study including a large sample size or a random sample is required to generalize our findings. Moreover, although we hypothesized that regular physical activity would enhance students’ social problem-solving ability, our study was cross-sectional in design. A longitudinal study is required to confirm the assumed direction of the causal association studied here.

We found a statistically significant, positive linear association between physical activity and social problem-solving ability after adjusting for sex. Because participants who exercised were less likely to be women than those who did not exercise (Table 1), we conducted stratified analyses to examine whether the association between physical activity and social problem-solving ability was dependent on sex. In consequence, we found that higher SPSI-R was more frequently observed among participants who exercised regularly, irrespective of the sex.

The present study had the following limitations. First, we considered age in years, sex, and living alone as potential confounding factors. However, because the questionnaire did not include socioeconomic status, a family history of mental disorders, and having a part-time job, we did not control for these factors. A further study controlling for these factors will be required. Second, the present study may have information bias in regards to physical activity, because our questionnaire did not include metabolic equivalents (METs) or frequency per week as indicators of physical activity. We estimated the *p* value for the linear trend using the five possible responses regarding physical activity level as a continuous variable (the responses were: “do not exercise, and never think about doing,” “do not exercise but intend to start,” “try to exercise but do not,” “exercise but not regularly,” and “exercise regularly”). Therefore, further studies using METs or frequency per week as a proxy for the amount of physical activity undertaken will be required. Third, because the response rate was low (6.8%), the participants may not have been representative of the source population of Tohoku Fukushi University students. Our study participants were more likely to engage in regular physical activity than average among all Japanese aged 20 to 29. Therefore, this low response rate should be considered when interpreting the study results. Moreover, a form of selection bias may have occurred wherein participants who were interested in physical activity or a healthy lifestyle may have been more likely to participate in the study.

## Conclusions

This study investigated the association between attitude and practice of physical activity and social problem-solving ability among Japanese university students. We found that higher SPSI-R could be more frequently observed among participants who exercised regularly, as compared to those who answered “I do not practice physical activity, and I never think about doing so”. In addition, there was a positive linear association between physical activity and higher social problem-solving ability scores. The present findings suggest that regular physical activity, or an intention to start physical activity, may function as effective strategies for improving social problem-solving ability, which may subsequently help in improving university students’ levels of mental health.

## Additional file

Additional file 1:
**Table S1.** Multivariate RR and 95% CI of higher SPSI-R and subscales according to physical activity. (DOCX 24 kb)

## Abbreviations

AS: Avoidance style
CI: Confidence interval
DM: Decision making
GAS: Generation of alternative solutions
ICS: Impulsivity/carelessness style
MET: Metabolic equivalent
NPO: Negative problem orientation
OR: Odds ratio
PDF: Problem definition and formulation
PPO: Positive problem orientation
RPS: Rational problem solving
RR: Rate ratio
SD: Standard deviation
SIV: Solution implementation and verification
SPSI-R: Social Problem-Solving Inventory–Revised
