# Supplementary material for: Attitude and practice of physical activity and social problem-solving ability among university students
Source: Environ Health Prev Med. 2017 Apr 4;22:18. doi: 10.1186/s12199-017-0625-8 (PMC5664572; doi:10.1186/s12199-017-0625-8)
Supplement: Additional file 1: — Table S1. Multivariate RR and 95% CI of higher SPSI-R and subscales according to physical activity. (DOCX 24 kb) [file 12199_2017_625_MOESM1_ESM.docx]

Additional file 1: Table S1. Multivariate RR and 95% CI of higher SPSI-R and subscales according to physical activity.

|  | Physical activity | | | | | P value for linear trend |
| --- | --- | --- | --- | --- | --- | --- |
|  | Do not exercise | | | Exercise but not regularly | Exercise regularly |  |
|  | And never think about doing so | But intend to start | Try to exercise |  |  |  |
| No. of subjects | 20 | 43 | 55 | 35 | 32 |  |
| SPSI-R |  |  |  |  |  |  |
| No. of subjects with higher SPSI-R | 6 | 20 | 23 | 20 | 24 |  |
| Crude RR (95% CI) | 1.00 (Ref.) | 1.55 (0.62–3.86) | 1.39 (0.57–3.42) | 1.90 (0.76–4.74) | 2.50 (1.02–6.12) | 0.02 |
| Multivariate adjusted RR (95% CI) | 1.00 (Ref.) | 1.57 (0.63–3.91) | 1.36 (0.55–3.36) | 1.78 (0.71–4.49) | 2.31 (0.93–5.74) | 0.05 |
|  |  |  |  |  |  |  |
| PPO |  |  |  |  |  |  |
| Multivariate adjusted RR (95% CI) | 1.00 (Ref.) | 1.02 (0.48–2.15) | 0.95 (0.45–1.97) | 0.93 (0.41–2.07) | 1.20 (0.55–2.63) | 0.70 |
| NPO |  |  |  |  |  |  |
| Multivariate adjusted RR (95% CI) | 1.00 (Ref.) | 0.91 (0.47–1.73) | 0.87 (0.46–1.64) | 0.70 (0.34–1.46) | 0.54 (0.24–1.20) | 0.09 |
| RPS |  |  |  |  |  |  |
| Multivariate adjusted RR (95% CI) | 1.00 (Ref.) | 0.83 (0.38–1.81) | 0.97 (0.47–2.00) | 0.89 (0.40–1.99) | 1.38 (0.65–2.96) | 0.25 |
| PDF |  |  |  |  |  |  |
| Multivariate adjusted RR (95% CI) | 1.00 (Ref.) | 1.11 (0.53–2.32) | 1.08 (0.53–2.22) | 0.85 (0.37–1.91) | 1.28 (0.60–2.77) | 0.75 |
| GAS |  |  |  |  |  |  |
| Multivariate adjusted RR (95% CI) | 1.00 (Ref.) | 1.08 (0.51–2.28) | 0.97 (0.46–2.01) | 1.15 (0.53–2.49) | 1.45 (0.68–3.09) | 0.27 |
| DM |  |  |  |  |  |  |
| Multivariate adjusted RR (95% CI) | 1.00 (Ref.) | 1.01 (0.44–2.32) | 1.01 (0.45–2.27) | 1.69 (0.75–3.82) | 1.73 (0.76–3.92) | 0.04 |
| SIV |  |  |  |  |  |  |
| Multivariate adjusted RR (95% CI) | 1.00 (Ref.) | 0.71 (0.35–1.43) | 0.89 (0.47–1.70) | 0.93 (0.46–1.88) | 1.15 (0.58–2.30) | 0.32 |
| ICS |  |  |  |  |  |  |
| Multivariate adjusted RR (95% CI) | 1.00 (Ref.) | 1.12 (0.57–2.19) | 0.98 (0.50–1.90) | 0.79 (0.37–1.67) | 0.71 (0.32–1.55) | 0.17 |
| AS |  |  |  |  |  |  |
| Multivariate adjusted RR (95% CI) | 1.00 (Ref.) | 0.86 (0.44–1.70) | 0.81 (0.42–1.56) | 0.77 (0.37–1.58) | 0.62 (0.29–1.34) | 0.22 |
| Abbreviations: *AS* avoidance style, *CI* confidence interval, *DM* decision making, *GAS* generation of alternative solutions, *ICS* impulsivity/carelessness style, *NPO* negative problem orientation, *PDF* problem definition and formulation, *PPO* positive problem orientation, *RPS* rational problem solving, *RR* rate ratio, *SIV* solution implementation and verification, and *SPSI-R* Social Problem-Solving Inventory–Revised.  The outcomes were higher for SPSI-R, PPO, NPO, RPS, PDF, GAS, DM, SIV, ICS, and AS.  Multivariate RR were adjusted for age in years (continuous variable), sex (men or women), and living alone (yes or no).  The p value for the linear trend was estimated using the five categories of physical activity as a continuous variable. | | | | | | |
